# Square root law model for the delivery and intestinal absorption of drugs: a case of hydrophilic captopril

**DOI:** 10.1080/10717544.2021.1960929

**Published:** 2021-08-06

**Authors:** Valentina Anuta, Constantin Mircioiu, Victor Voicu, Ion Mircioiu, Roxana Sandulovici

**Affiliations:** a Department of Physical and Colloidal Chemistry, Faculty of Pharmacy, “Carol Davila” University of Medicine and Pharmacy, Bucharest, Romania; b Doctoral School, “Carol Davila” University of Medicine and Pharmacy, Bucharest, Romania; c Department of Biopharmacy and Pharmacokinetics, Titu Maiorescu University, Bucharest, Romania; d Department of Applied Mathematics and Biostatistics, Titu Maiorescu University, Bucharest, Romania

**Keywords:** Modeling the absorption rate, diffusion equation, release from reservoirs, CPT absorption, square root law of absorption

## Abstract

The *in vivo* release and absorption of drugs are dependent on the interplay between many factors related to compound, formulation, and physiological properties. The mathematical models of oral drug absorption attempt to strike a balance between a complete description that takes into consideration as many independent factors as possible, and simple models that operate with fewer parameters, based mainly on critical factors. The latter models are by far more robust and easier to apply to predict the extent and sometimes even the rate of absorption. The present paper attempted to develop a simple model to describe the time course of absorption of the hydrophilic drug captopril (CPT) at the early phases of absorption, with implications mainly in the induction and early stages of achieving its therapeutic effect. As a phenomenological model, the instantaneous release of CPT was considered in the gastrointestinal fluid, leading to a constant drug concentration for a prolonged time, followed by a ‘long path diffusion’ inside the intestinal wall and a very low concentration at the interface intestinal wall-blood. These conditions regarding CPT concentration were translated into initial and boundary mathematical conditions for the diffusion equation in the intestinal wall. The solution of the diffusion equation led in the end to a square root law describing the dependence between the fraction of the drug absorbed and time. The model was successfully applied to data obtained in five bioequivalence studies: three comparing plasma levels achieved after the administration of a single dose of CPT 50 mg, one evaluating CPT pharmacokinetics after a 100 mg dose, and a fifth comparing CPT pharmacokinetics of two fixed-dose combinations of CPT 50 mg and hydrochlorothiazide 25 mg.

## 1. Introduction

Release in the gastrointestinal tract and the intestinal absorption of drugs into the bloodstream are the first steps in the pharmacokinetics of orally administered drugs. The rate and extent of absorption are essential factors in pharmacokinetic-pharmacodynamic coupling (Mager et al., 2003; Crommelin et al., 2008), and finally in the balance between therapeutic efficacy and safety of drugs. Research and development of a new drug include answering questions about how the respective drug is released, absorbed, metabolized, distributed, and eliminated.

Factors affecting drug release and absorption can be divided into three different categories. The first category is represented by the structure of the pharmaceutical formulation and a multitude of physicochemical properties of the active compounds, including pKa (Wan and Ulander, 2006; Manallack, 2007), solubility in biorelevant media (Mircioiu et al., 2012; Preda et al., 2012), the partition coefficient (Perez et al., 2002), permeability in different segments of the gastrointestinal (GI) tract (Lozoya-Agullo et al., 2016; Chung and Kesisoglou, 2018; Vertzoni et al., 2019), particle size distributions (Hintz and Johnson, 1989), supersaturation (Hens, 2018), the substrate of transporters (González-Alvarez et al., 2007), the effect of interactions between poorly soluble drugs and surface-active agents (Mircioiu et al., 2013a), etc. However, it is essential to understand that this multitude of parameters is in fact a mathematical space with a smaller number of dimensions, as these parameters are not independent.

Anatomical and physiological conditions represent the second component of the drug-living body interaction. Factors, such as pH, peristalsis (Enobong et al., 2018), post-dose contractions (Bermejo, et al., 2018), physiological surfactants (Carmona-Ibanez et al., 1999), buffer capacity (Hens et al., 2017), accumulation and transfer of drugs and physiological surfactants at interfaces (Bermejo et al., 1999), gastric emptying (Chrenova et al., 2010), and enterohepatic circulation (Tvrdonova et al., 2009) significantly influence the rate and extent of absorption.

In terms of pharmaceutical technology, the inclusion of drugs in colloidal vectors, i.e. formulation as an immediate or extended-release drug, as well as the addition of surfactants and the formation of supramolecular structures (Miclea et al., 2010; Suta et al., 2013) are usual methods for improving bioavailability and to better manage pharmacokinetics. Mathematical models concerning absorption attempt to find a balance between a complete description that takes into consideration as many independent factors as possible, and simple models that operate with fewer parameters, based mainly on critical factors. Models of the first type are very complex and are associated with insurmountable difficulties in finding analytical expressions and the identification of parameters for particular cases. The second type of model leads to simple laws, but performance in fitting experimental data is sometimes reduced. Almost all models proposed until now have attempted to estimate the fraction of dose absorbed (FRA). This includes the pH partition model (Chanker, 1960), absorption potential models (Dressman et al., 1985; Macheras and Symillides, 1989), film models (Amidon et al., 1988), and macroscopic balance (Sinko et al., 1991).

Dynamic models have attempted to predict both the fraction of dose absorbed and the rate of drug absorption and to correlate them to pharmacokinetic models for predicting plasma concentration profiles. Such models are however much too complex, requiring extensive physicochemical and physiological information (Lin and Wong, 2017). There have been numerous attempts considering the factors listed above to be included in mathematical models, but this has led to increasingly cumbersome models, which have been very difficult to apply in practice, and fitting algorithms being generally unstable, i.e. sudden, great changes in the estimated parameters in the case of small changes in the input data.

This paper aimed at a simple model, with an analytical simple solution, restricted to a finite time (Macheras et al., 2018) to describe the time course of absorption of the CPT, deduced by deconvolution of its plasma levels in five bioequivalence studies.

## 2. Methods

### 2.1. *In vitro* release

Release experiments were performed using the USP basket apparatus (ERWEKA DT800 HH model) at a rotation speed of 50 rpm, as specified in the CPT tablet monography of USP32 (USP29-NF24). As a release medium, 900 ml of 0.01 N HCl was used. Samples with a volume of 2 ml were collected at 5, 10, 15, 20, and 30 min and then filtered through a 0.45 µm Teflon^®^ filter.

Dissolution studies were performed in the frame of five different bioequivalence studies. Experiments were run on 12 tablets of each formulation. The quantification of CPT was achieved by using a spectrophotometric method at *λ* = 205 nm.

### 2.2. Clinical study

A single dose of CPT was administered to subjects (*n* > 20) in five, cross-over, two-period, two sequence bioequivalence studies under fasting conditions, with a washout period of 6 days between period I and period II. Studies were approved by the Romanian National Ethics Committee and the Romanian National Medicines Agency.

Three studies, denoted as B, M, and L, compared plasma levels achieved after the administration of CPT (CPT) 50 mg. Another study, denoted S, compared the pharmacokinetics of CPT after a 100 mg dose. Because it is common to combine CPT with hydrochlorothiazide (HCT) in a single tablet, the fifth study compared two formulations containing a fixed-dose combination of 50 mg CPT and 25 mg HCT to test the effect of the pharmacokinetic interaction on CPT absorption.

Blood samples were collected before, and 0.5, 1, 1.5, 2, 2.5, 3, 4, 5, 6, 8, 10, 12, and 24 h after drug administration. Plasma levels of the active substance CPT were determined using a validated liquid chromatographic method (Medvedovici et al., 2009). Pharmacokinetic analysis of the active components was performed using non-compartmental methods and modeled using mono- and bicompartmental models. The estimation of the parameters of the compartmental models was performed using Pk Solver 2 software version 2.2.

### 2.3. Calculation of the time dependence of the absorption fraction

**Figure 1.**
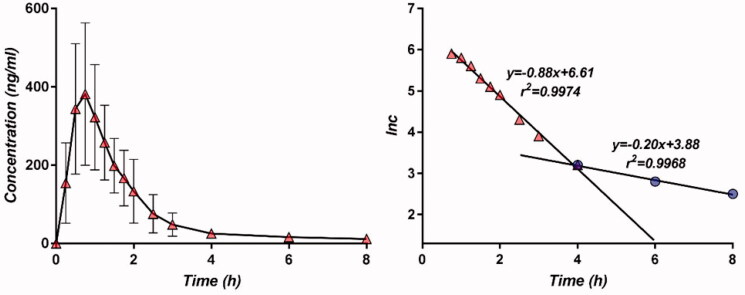
Concentration and logarithm of concentration as a function of time (study B, reference drug) after the time point of the maximum concentration.

The absorption fraction *FRA(t)* of CPT was deduced from its plasma levels. It was observed that, after the time of maximum concentration, plasma levels decreased in two consecutive monoexponential phases: one from the maximum concentration to 4 h and a second, slower release phase at the tail of the curve, corresponding to <5% of the area under the curve (Figure 1).

The slope of the first regression line was considered as ‘distribution constant’ and the second slope as an elimination constant, although their interpretation is more complex.

In a mass balance approach, similar to the Nelson Wagner method, it was considered that the absorbed quantity at time t is the sum of the quantity in the blood at moment t and the quantity that has disappeared from the blood via first-order kinetics, with a rate constant *k_d_*

(1) $$\mathrm{FRA}(t_{i})=\frac{V_{d}c(t_{i})+\int_{0}^{t_{i}}k_{d}V_{d}c(t)dt}{\int_{0}^{\infty }k_{d}V_{d}c(t)dt}=\frac{c(t_{i})+k_{d}AUC_{0-t_{i}}}{k_{d}AUC_{0-\infty }}$$

where *V_d_* is the distribution volume, $AUC_{0-t_{i}}$ is the area under the concentration-time curve of the drug from time 0 to time *t_i_* calculated from experimental data using the trapezoidal rule, and $AUC_{0-\infty }$ is the area under the concentration-time curve of the drug from time 0 to infinity.

## 3. The square root model of absorption from an infinite reservoir

The model supposes diffusion-controlled absorption and gives a solution for the equation

(2) $$\frac{\partial c(x,t)}{\partial t}=D\frac{\partial^{2}c(x,t)}{\partial x^{2}}$$

where *c*(*x,t*) is the concentration in the epithelial wall and *D* is the diffusion coefficient, in the following phenomenological conditions, translated into initial and boundary conditions (Table 1).

**Table 1.** Mathematical initial and boundary conditions and solution of the diffusion equation.

Intestinal fluid	Intestinal wall	Blood
Phenomenological condition	Phenomenological conditions	Phenomenological conditions
Instant release of CPT in intestinal fluid and set up of a constant concentration for a significant time interval.	Concentration of CPT in the intestinal wall at the interface with intestinal fluid is initially zero Concentration increases suddenly and remains constant at a value $c_{0}$ Diffusion of CPT in the wall is a ‘long path process’ such that at the interface of intestinal wall with the blood the concentration is very low and we can consider it zero	The blood is a ‘compartment,’ with same concentration at all points
Mathematical condition	Mathematical, initial and boundary conditions	Mathematical conditions
*c*(*x*,*t*) = constant	$x=0\;c(0,t)=c_{0}$t=0 c(x,0)=0 $\underset{x\to \infty }{\lim}c(x,t)=0$ The solution of the equation is: $c(x,t)=c_{0}\left(1-\mathrm{erf}\frac{x}{\sqrt{4Dt}}\right)$	*c*(*x*,*t*) = *c*(*t*)
	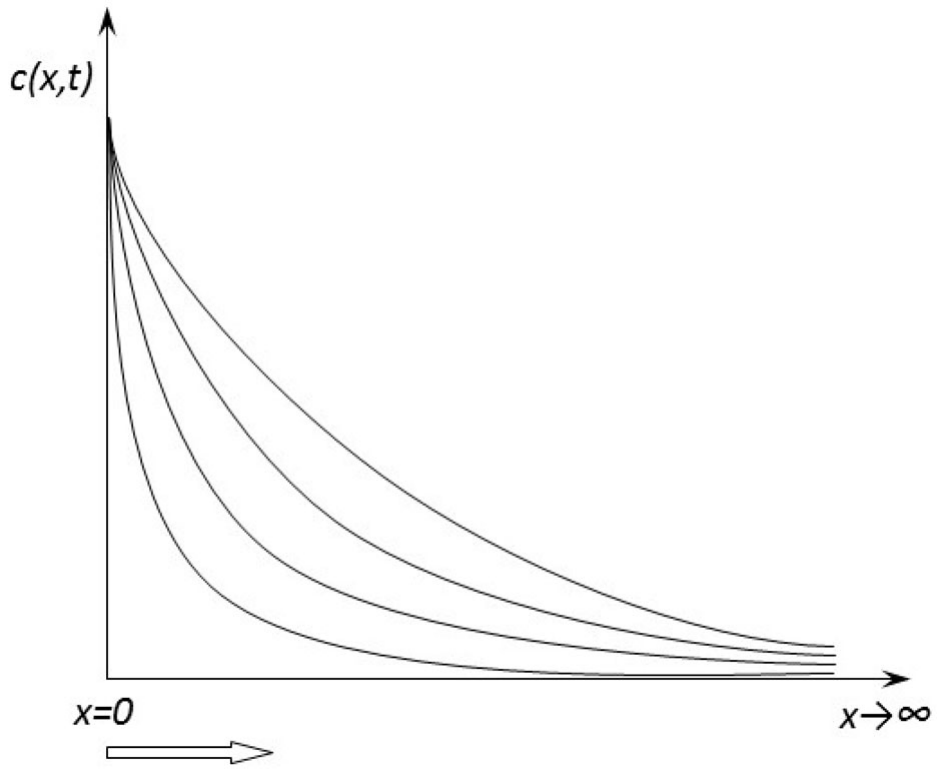	
	Flux: $J=-D\frac{\partial c}{\partial x}(0,t)$ Transferred amount *Q(t)* $\frac{Q(t)}{A}=\frac{2c_{0}}{\sqrt{\pi }}\sqrt{Dt}$	

1. Since the drug is hydrophilic, its release *in vivo* is supposed to be very rapid, similar to release *in vitro*. Intestinal fluid is considered as a compartment, which means a spatial homogeneous concentration. Since permeability is low, the intestinal wall act as a barrier to transport so that the concentration remains high and relatively constant for a significant time. Under these conditions, intestinal fluid can be considered mathematically as an infinite reservoir with a constant concentration – *c_0_*, similar to a thermostat in heat transfer theory. This corresponds to the boundary condition for the diffusion equation:

(3) $$x=0\;c(0,t)=c_{0}$$

2. If we suppose that the active substance is not present in the intestinal wall before absorption, the mathematical ‘initial condition’ for the differential equation of diffusion is:

t=0 c(x,0)=0

3. The diffusion domain is considered the intestinal wall.

4. Considering the thickness of the intestinal wall as a long pathway for diffusion, it is possible to consider mathematically diffusion in a semi-infinite medium with the condition at the infinite frontier:

$$\underset{x\to \infty }{\lim}c(x,t)=0$$

The solution of the diffusion equation for the concentration inside the intestinal wall with these initial and boundary conditions can be obtained by its transformation in an ordinary linear equation after application of the Laplace transform to *c*(*x*,*t*) as a function of *t*. Solving the obtained differential equation and applying the inverse Laplace transform gives (see Appendix A) the solution:

$$c(x,t)=c_{0}\left(1-\mathrm{erf}\frac{x}{\sqrt{4Dt}}\right)$$

where erf is the ‘error function’:

(4) $$\mathrm{erf}(x)=\frac{2}{\sqrt{\pi }}\int_{0}^{x}e^{-u^{2}}du.$$

The main problem proposed in this model is to calculate the kinetics of transfer across the internal face of the intestinal wall, more exactly the quantity of the active component released from the intestinal fluid as a function of time. Emphasis is placed on the rate of transfer, FRA(*t*). For this aim, let us calculate the flux *J*(*t*) (quantity transferred in a time unit *dm*/*dt* across an area *A*) of the drug across the intestine face of the intestinal wall (*x* = 0).

We applied Fick’s first law:

(5) $$J=\frac{1}{A}\frac{dm}{dt}=-D\frac{\partial c}{\partial x}(0,t)$$

If we denote the combined variable $\frac{x}{\sqrt{4Dt}}$ by *y*, then the flux *J* will be:

(6) $$J=-D\frac{\partial c}{\partial x}(0,t)=-D\frac{\partial c}{\partial y}\frac{\partial y}{\partial x}=Dc_{0}\frac{2}{\sqrt{\pi }}e^{-\frac{x^{2}}{4Dt}}\frac{1}{\sqrt{4Dt}}(0,t)=Dc_{0}\frac{1}{\sqrt{\pi Dt}}$$

It was therefore found that the flux of the drug across the interface is proportional to the inverse of the square root of time. We can further compute the quantity *Q*(*t*) of drug transferred after time t, across the interface *x* = 0:

(7) $$\frac{Q(t)}{A}=\int_{0}^{t}\mathrm{Jdt}=\int_{0}^{t}\frac{1}{A}\frac{dm}{dt}dt=\int_{0}^{t}Dc_{0}\frac{1}{\sqrt{\pi Dt}}dt=\frac{2c_{0}}{\sqrt{\pi }}\sqrt{Dt}$$

The result indicates that the quantity of active substances which left the intestinal content and can be considered as having entered the bloodstream is a linear function of the square root of time. Hence, if the representation of the absorbed fraction as a function of the square root of time is a straight line, we indicate that the model is applicable.

## 4. Results

### 4.1. *In vitro* release and pharmacokinetics

**Figure 2.**
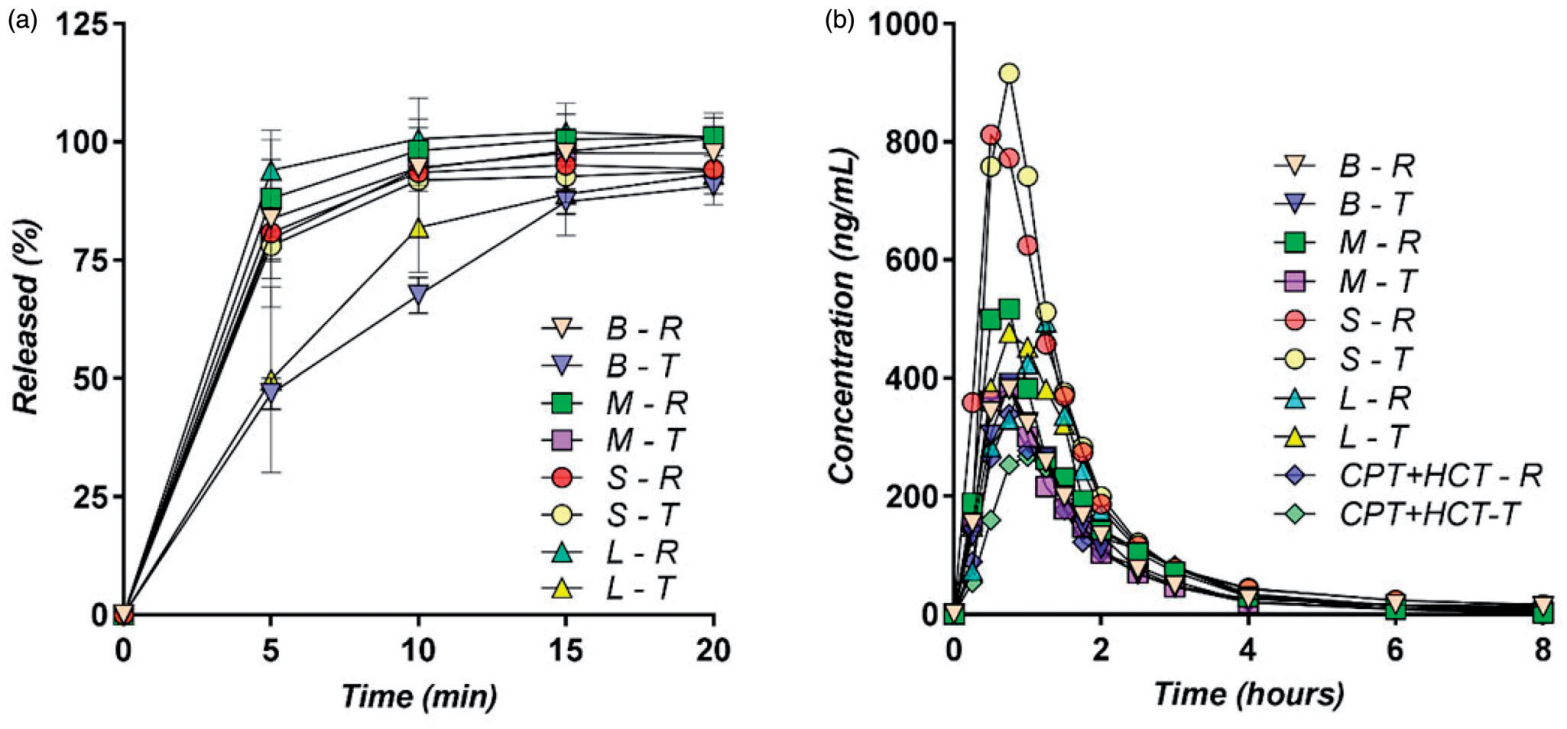
Dissolution profiles for the CPT formulations in the B, M, L, and S studies (a) and plasma CPT levels in the evaluated pharmacokinetic studies (b); R: reference drug; T: tested drug.

*In vitro* release was very rapid from all 10 formulations. Two tested formulations had a somewhat slower release, but at 20 min, the release was more than 80% complete, regardless of the formulation (Figure 2(a)). A possible square root law for *in vitro* release appeared in the case of the two formulations with the slower release.

Following this result, it was reasonable to think that, at least for a period of time, the concentration of CPT in the intestine is high and constant.

Pharmacokinetic was similar for all tested formulations (Figure 2(b)). The two higher mean maximum concentrations corresponded to the double dose (100 mg) and the two somewhat lower concentrations concerned the CPT + hydrochlorothiazide tablets. Pharmacokinetics were proportional to the administered dose, the maximum concentration, and the area under the curve, increasing by approximately double in the case of the 100 mg dose in comparison to the 50 mg dose. Absorption and elimination were very rapid, and the area under the curve at 4 h (AUC_0–4 h_) was more than 95% of the total area extrapolated to infinity.

**Figure 3.**
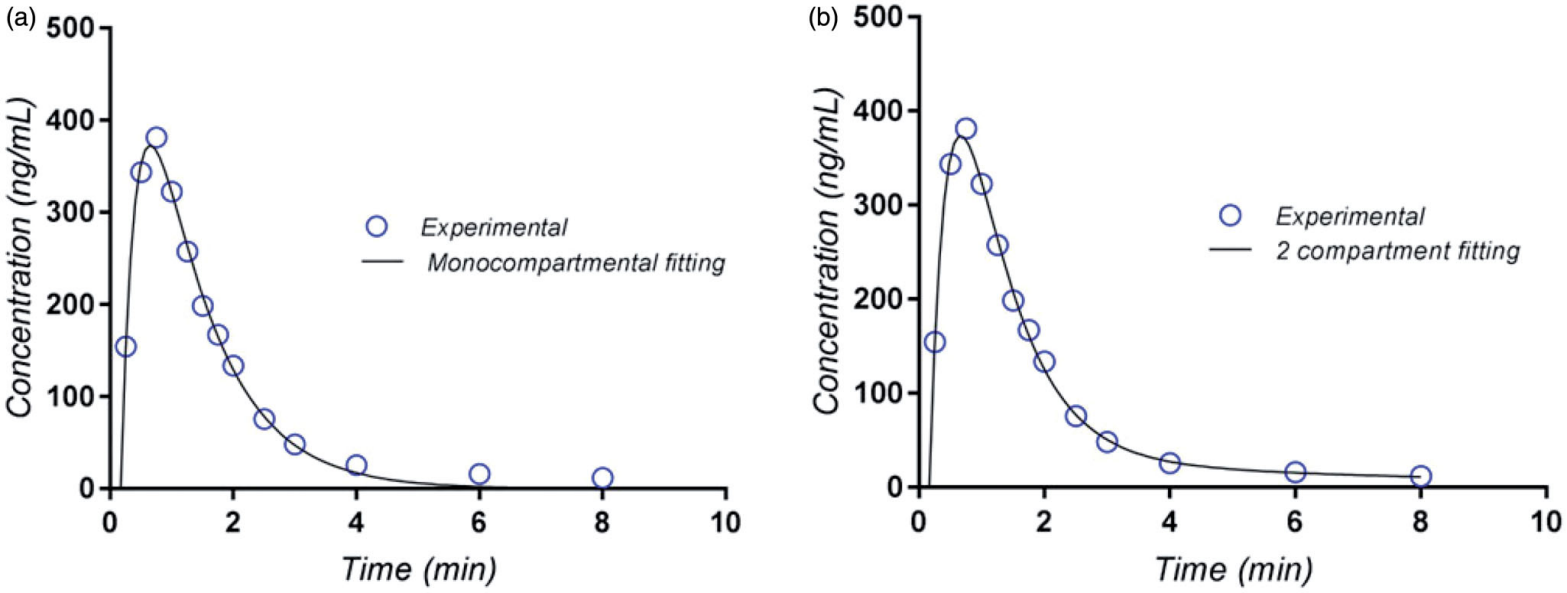
Monocompartmental (a) and bicompartmental (b) modeling of CPT pharmacokinetics, formulation B.

Fitting of plasma levels for the 0–4 h time interval led to similar results for both the bicompartmental and monocompartmental models. An improvement occurred in the two-compartment model in the elimination phase, as illustrated for the reference drug in study B in Figure 3, but the improvement after increasing the number of parameters was not statistically significant and refers to the last two points, which correspond to <1% of the area under the curve (Prasacu et al., 2009; Sandulovici et al., 2009).

The calculated elimination constant differed for the two models. The constant that resulted from the monocompartmental model was close to the ‘distribution constant’ calculated from analysis of the regression line fitting the data on the tail of the plasma level curve, as presented above. In fact, a second compartment, following the hydrophilicity of CPT is not the lipid compartment, but rather disulfide conjugation products (Savu et al., 2016). The final elimination constant associated with small amounts of residual CPT could be connected with the partial reversibility of conjugation reactions.

### 4.2. Square root modeling of FRA(t)

**Figure 4.**
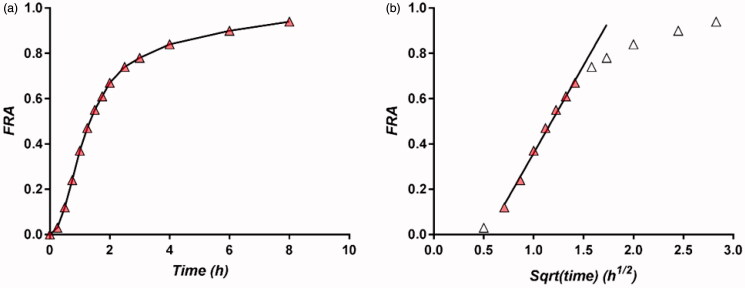
Selection of the domain of estimation of FRA as a linear regression with the square root of time: FRA as function of time (a), FRA as function of square root of time (b).

The method for calculating FRA curves as a function of time is presented in Figure 4. In all cases, a small lag time of ∼5 min appeared, so the zero point was not considered.

A saturation of the FRA(t) curve appeared as a rule after reaching the value of 0.8. The square root model was considered for FRA from the first measuring point to the observed saturation of absorption, as a rule around 0.8, since it was considered that the phenomenological and mathematical hypotheses are less valid for greater values, although in several cases the model worked even after this threshold, the entire process being diffusion controlled. This is less usual but not a singular case (Gusea, 2006). A threshold of 0.7 was established based on experience from the last 50 years, in the case of the Higuchi square root law describing the release of active components from solid and semisolid formulations as well as release from almost all supramolecular systems. It was suggested that, in fact, many applications of the square root release law are not connected with the Higuchi model, but rather with release from an infinite reservoir into a semi-infinite medium .

**Figure 5.**
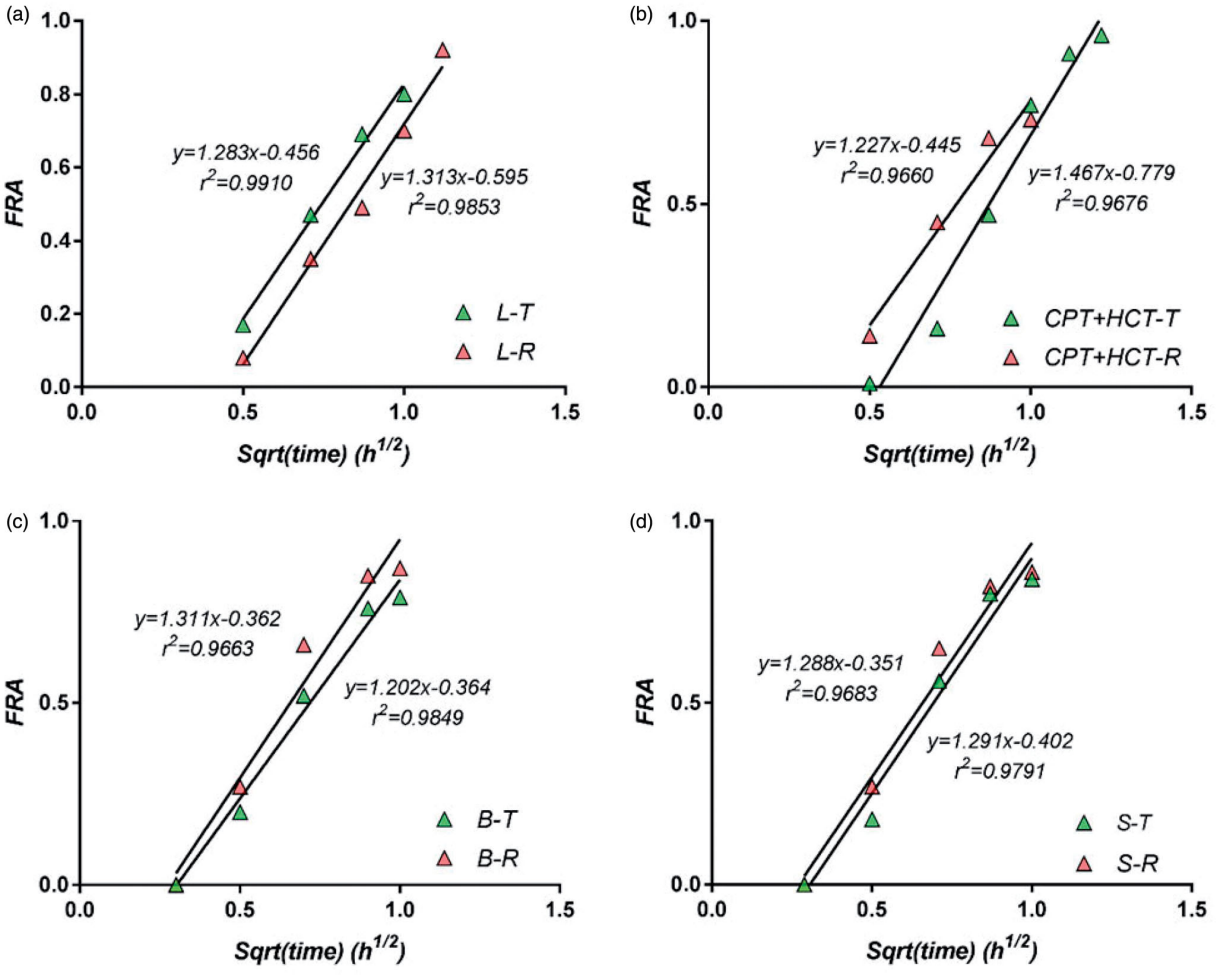
Correlation between FRA and square root of time, in studies B (a), L (b), S (c), and CPT + HCT (d); R: reference drug; T: tested drug.

In the fittings, a time lag of ∼5 min was considered. Since in all studies the formulations proved to be bioequivalent, the calculated absorption fractions were analyzed and fitted with the square root of time in parallel. The results of fitting the data to the theoretical model for studies B, L, S, and CPT + HCT are presented in Figure 5.

The best results were obtained in study L for both the reference and tested drug, as the correlation was very good (correlation coefficients above 0.99). The slopes of the linear regression were the same for both formulations. In study B, the correlations were good enough and reached values in the neighborhood of FRA = 0.9. The slopes corresponding to the two formulations were slightly different (1.20 and 1.31).

In study S, with double the dose of CPT, the results were not as good as in the case with lower doses. It was noteworthy that the correlation reached a fraction >0.8. In study M (data not shown), the results were very similar to those of study B. The evaluation of joint data on the reference and tested drugs did not significantly change the results.

**Figure 6.**
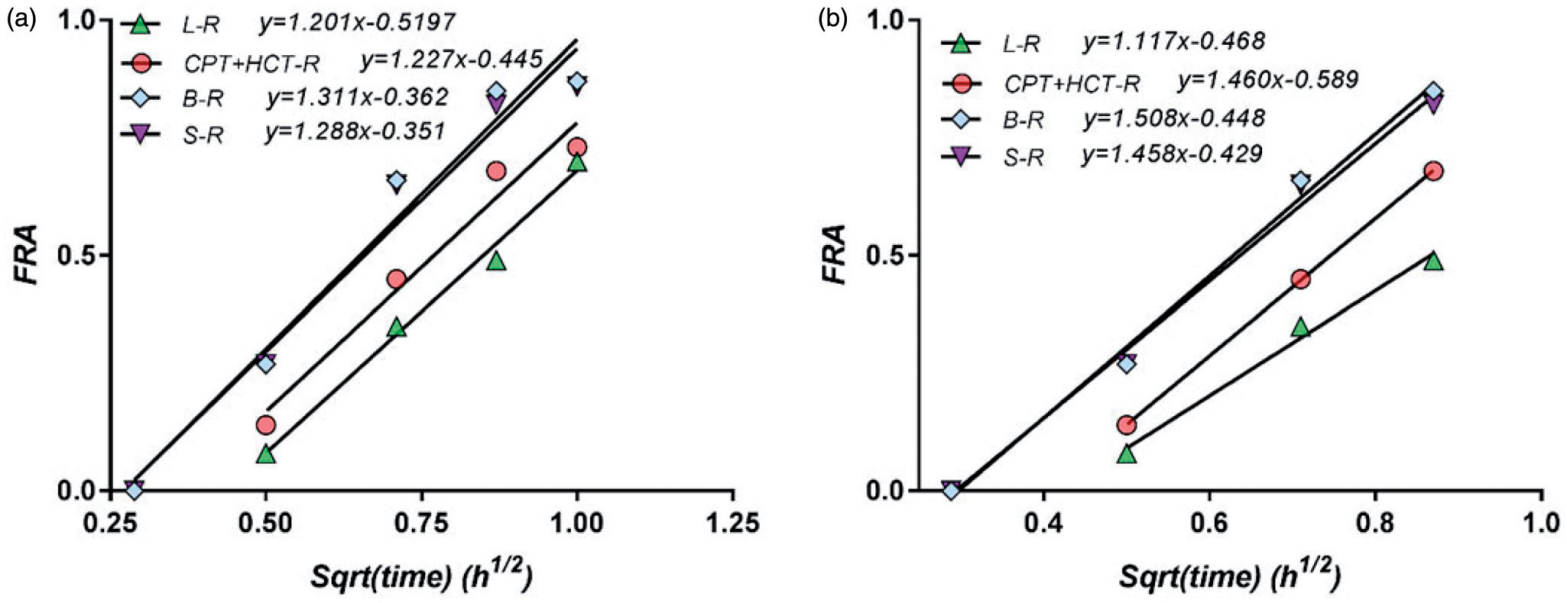
Correlation between FRA and square root of time for the reference formulation in four different studies within the first hour (a) and for the first 45 min (b).

Some of the differences between pharmacokinetic experiments could arise from differences in the formulation and differences in the characteristics of the healthy volunteers (Mircioiu et al., 2005). Since the reference drug was the same in all experiments, to estimate the effect of the differences between lots, we represented the FRA and its dependence on the square root of time in the case of the reference formulation in the different bioequivalence studies. As can be seen in Figure 6, the regression lines were approximately parallel, so the influence of the demographic characteristics of the subjects is not a critical factor.

Last but not least, the performance of the model was also dependent on the time interval of the pharmacokinetic data. As presented above, the first square root time approximated reliable was 0.5, i.e. 15 min. In all cases, apparent saturation appeared after 1 h. Since the time of maximum concentration was 40 min, the fitting performance was also checked in this time interval. As can be seen in Figure 5, restriction to Tmax led to a practically perfect fit.

## 5. Discussion

Almost all mathematical models attempt to predict the fraction of drug absorbed (FRA) as a function of different parameters. Our model tries to predict both FRA and its kinetics, starting from drug concentration in the intestinal wall as a function of both time and space.

The first models of this type, describing the diffusion of drugs in space and time, were inspired by models of heat transfer. Simultaneous longitudinal flow and diffusion in the intestine to the place of absorption was proposed 40 years ago in a classical paper (Ni et al., 1980), which considered release from an infinite drug reservoir along the intestine, taken as a hollow cylinder. The model solved the diffusion equation under particular initial and boundary conditions, obtaining an analytical solution for *c*(*x*,*t*) which allowed the prediction of the ‘kinetics of appearance of the unabsorbed drug at the end of the intestinal segment.’

Also, an analytical solution was obtained later, ‘assuming that the stomach can be considered as an infinite reservoir with a constant output rate with respect to concentration and volume’ (Yu, 1996). These authors solved the diffusion equation using similar initial and boundary conditions to those used in our paper, using the Laplace transform method, based on the theory of heat transfer (Carslaw and Jaeger, 1959). In fact, this was simpler than the contour integral method used previously and represents a generalization of the Fourier, Laplace, and Fick models from 200 years ago.

It is worth noting the significant fact that, in our model, the space of diffusion is the intestinal wall, not the stomach or the intestine. Other authors have also considered passive membrane diffusion processes, where molecules pass across membranes driven by concentration gradients in the absorption of many drugs (Lennernäs, 2007; Sugano et al., 2010). Other models are considered ‘dynamic,’ in the sense of considering both the time and space dependence of the concentration in a given domain. The mixing tank model has been developed and utilized to simulate oral absorption phenomena. This approach considers the gastrointestinal tract as one or more serial mixing tanks with linear transfer kinetics. Each tank is considered well-mixed and having a uniform concentration (Dressman et al., 1984; Dressman and Fleisher, 1986). In the compartmental absorption and transit model (CAT) (Yu, 1996), the process of the passing of drugs through the small intestine was viewed as flow through a series of segments. Each segment was described as a single compartment with linear transfer kinetics. The ACAT model (Agoram, 2001) was developed based on the CAT model to include first-pass metabolism and colon absorption.

If we consider *x* as a discrete variable taking values from 1 to 7, we can speak about a *c*(*x*,*t*) concentration in the above models, but the interpretation is forced and the diffusion equation is lost. However, consideration of intestinal fluid as a compartment with a rapidly obtained homogeneous concentration is an implicit hypothesis of all pharmacokinetic models for the immediate release of oral dosage forms. A consequence of this hypothesis, in conjunction with the hypothesis of intercompartmental linear transfer in concentration leads to the exponential absorption model, which remains the simplest and most largely accepted model.

In the case of the ingestion of food during drug *in vivo* dissolution, a critical factor in the determination of the drug concentration in the intestinal fluid is gastric emptying and intestinal peristalsis (Oberle and Amidon, 1987). The hypothesis of a constant reservoir in the intestine ceases to be valid after a certain time as the concentration decreases due to absorption. However, since the absorption of hydrophilic drugs is poor, this remains for a relevant time, approximately constant.

The square root law is not unusual in biopharmacy. It was used to assess the transfer of toxins through the skin (Mircioiu et al., 2013b) and in the case of release from cubosomes as a reservoir (Paolino et al., 2019). The law is similar to the Higuchi law concerning release from solid and semisolid drug formulations, but it was deduced under very different conditions. In the case of CPT, it seems that the hypothesis is valid. On the other hand, the quantity of CPT distributed in a hypothetical deep compartment is neglected in our model. The model for CPT pharmacokinetics, as other authors have reported before, appears to be a bicompartmental one (Pereira et al., 1996).

Critical phenomena associated with bile salts after food intake can significantly influence the interfacial transfers of active substances (Pahomi et al., 2012) and particularly transfer from intestinal fluid to the intestinal wall. Accordingly, the model parameters could be correlated with temporal modifications in the composition of the intestinal fluid.

Finally, many influences could occur due to numerous factors interfering with the initial and boundary conditions considered by the model. Despite these complications, in the case of all five bioequivalence studies of CPT analyzed in this paper, the law worked acceptably well.

A complete presentation of the method of solving differential equations of diffusion, presented in the Appendix, provides evidence of the essential role of the initial and boundary conditions coming from phenomenological conditions. The importance of understanding this correspondence is the prevention of attempts to apply models under conditions incompatible with the conditions of deriving the respective models; this mistake is frequently seen in the analysis of release from all supramolecular systems.

## 6. Conclusions

Consideration of the absorption of an active drug component as diffusion from the intestine as an infinite reservoir in the intestinal wall as a semi-infinite medium led to a square root law for the dependence of the absorbed fraction on time.

A mass balance model for the amount of drug in the blood led to a formula for the calculation of the absorbed fraction starting from plasma level data, similar to that of Nelson-Wagner but based on the distribution constant instead of the elimination constant.

The square root law proved to be applicable in the case of ten different pharmacokinetic experiments concerning CPT 50 and 100 mg and two regarding CPT plus hydrochlorothiazide for ∼80% absorption of the available drug.

Prediction of first, the major part of absorption, is considered significant in the context of prediction of induction and the first part of the effect.

## Appendix A. The solution of the diffusion equation for release from a reservoir with constant concentration in a semi-infinite medium

The next mathematical solution of the diffusion equation under specified initial and boundary conditions actually comes from the mathematical theory of heat transfer from a thermostat to a semi-infinite medium. The identification of different pharmaceutical reservoir systems with a thermostat represents a natural extension. The importance of the demonstration is that it highlights the essential fact that the solution is valid and intrinsically connected with the initial and boundary conditions translating the phenomenological characteristics of the system.

Let us solve the diffusion equation

$$\frac{\partial c(x,t)}{\partial t}=D\frac{\partial^{2}c(x,t)}{\partial x^{2}}$$

under conditions imposed by the proposed model:

$$x=0\;c(0,t)=c_{0}$$

t=0 c(x,0)=0

$$\underset{x\to \infty }{\lim}c(x,t)=0$$

We apply the Laplace transform to concentration as function of time:

$$L\left(c\left(x,t\right)\right)=\int_{0}^{\infty }c\left(x,t\right)e^{-pt}dt=C(x,p)$$

where we denoted the image function as *C*(*x*,*p*).

Starting from definition of the Laplace transform, we easily obtain:

$$L\left(\frac{\partial c}{\partial t}\right)=L\left(c\prime \right)=pC(x,p)-c(x,0)=pC(x,p)\;\text{and}\;L\left(\frac{\partial^{2}c}{\partial x^{2}}\right)=\frac{\partial^{2}C}{\partial x^{2}}.$$

Applying the transform to both members of the equation, it results:

$$pC(x,p)=D\frac{\partial^{2}C}{\partial x^{2}}$$

Considering C(x,p) as a function of *x*, we obtained the ordinary differential equation:

pC¨−pC=0

Looking for solutions of the form $e^{rx}$ (Euler method), the characteristic equation is obtained:

$$x^{2}-\frac{p}{D}=0\text{ with two solutions}:\sqrt{\frac{p}{D}}\mathrm{and}-\sqrt{\frac{p}{D}}.$$

Consequently, the general solution can be written in the form:

$$C\left(x,p\right)=\alpha e^{-\sqrt{\frac{k}{D}}x}+\beta e^{\sqrt{\frac{k}{D}}x}$$

where α and β are constants, which can be identified using the initial and boundary conditions.

Since $(0,t)=c_{0}$ :

$$C\left(0,p\right)=\int_{0}^{\infty }c\left(0,t\right)e^{-pt}dt=\int_{0}^{\infty }c_{0}e^{-pt}dt=\frac{c_{0}}{-p}{e^{-pt}|}_{0}^{\infty }=\frac{c_{0}}{p}$$

But, on the other hand

$$C\left(0,p\right)=\alpha e^{-\sqrt{\frac{k}{D}}0}+\beta e^{\sqrt{\frac{k}{D}}0}=\alpha +\beta .$$

Further:

$$\underset{x\to \infty }{\lim}c(x,t)=0\Rightarrow \underset{x\to \infty }{\lim}\int_{0}^{\infty }c\left(x,t\right)e^{-pt}dt=\int_{0}^{\infty }\underset{x\to \infty }{\lim}c\left(x,t\right)e^{-pt}dt=\int_{0}^{\infty }0e^{-pt}dt=0.$$

But,

$$\underset{x\to \infty }{\lim}\int_{0}^{\infty }c\left(x,t\right)e^{-pt}dt=\underset{x\to \infty }{\lim}C\left(x,p\right)=\underset{x\to \infty }{\lim}\left(\alpha e^{-\sqrt{\frac{k}{D}}x}+\beta e^{\sqrt{\frac{k}{D}}x}\right),$$

and the last limit is zero only in the case β=0.

Since it was above established that

$$\alpha +\beta =\frac{c_{0}}{p},$$

$$\alpha =\frac{c_{0}}{p}$$

and

$$C(x,p)=\frac{c_{0}}{p}e^{-\sqrt{\frac{p}{D}x}}.$$

From tables of original functions corresponding to different image functions (found in all engineering handbooks)

$$c(x,t)=c_{0}\left(1-\mathrm{erf}\frac{x}{\sqrt{4Dt}}\right),$$

where erf is the ‘error function’ $\mathrm{erf}x=\frac{2}{\pi }\int_{0}^{x}e^{-u^{2}}du.$
